# Decoupling bioaccumulation and trophic transfer: nanoatrazine accumulates in microalgae but shows limited transfer to zooplankton

**DOI:** 10.1007/s10661-026-15831-y

**Published:** 2026-09-01

**Authors:** Ana Paula Andrade Braga, José Henrique Vallim, Mara Denise Luck Mendes, Vítor Oliveira Levanteza, Márcia Regina Assalin, Marley Mendonça Tavares, Leonardo Fernandes Fraceto, Anderson do Espírito Santo Pereira, Vera Lucia Scherholz Salgado de Castro

**Affiliations:** 1 Laboratory of Ecotoxicology and Biosafety, Embrapa Environment, Jaguariúna, São Paulo, Brazil; 2 Department of Environmental Engineering, Institute of Science and Technology of Sorocaba, São Paulo State University (UNESP), Sorocaba, São Paulo, Brazil

**Keywords:** Nanopesticides, Aquatic food web, Nanoecotoxicology, *Raphidocelis subcapitata*, *Daphnia magna*

## Abstract

Nanoformulations of pesticides have been proposed as a strategy to improve agricultural efficiency while potentially reducing environmental contamination. However, little is known about the bioaccumulation and trophic transfer of nanopesticides in aquatic food webs. In this study, the bioaccumulation of poly(ε-caprolactone)-encapsulated nanoatrazine (nATZ-PCL) was evaluated in the microalga *Raphidocelis subcapitata*, as well as its potential transfer to the zooplanktonic consumer *Daphnia magna* through dietary and direct exposure pathways. Physicochemical characterization demonstrated that the nATZ-PCL presented relative stability under most tested conditions, although aggregation behavior was observed in the algal culture medium over time. Atrazine accumulation was detected in *R. subcapitata*, particularly under EC_25_ exposure conditions in the absence of natural organic matter (NOM). In contrast, atrazine was not detected above the analytical detection limit in *D. magna* following either dietary exposure with pre-exposed algae or direct exposure in reconstituted medium. The results suggest that nanoparticle aggregation, reduced bioavailability, limited assimilation efficiency, and depuration processes may have restricted the trophic transfer of nATZ-PCL to the primary consumer. These findings demonstrate that bioaccumulation in primary producers does not necessarily imply efficient transfer across aquatic trophic levels and contribute to a better understanding of the environmental behavior of nATZ-PCL in aquatic systems.

## Introduction

Atrazine is one of the most widely used herbicides worldwide, particularly applied in maize, sugarcane, and sorghum crops to control broadleaf and grassy weeds (WHO & FAO, 2019). Due to its high-water solubility and environmental persistence, atrazine frequently reaches aquatic ecosystems through surface runoff, leaching, and atmospheric deposition (Deng et al., 2024). Once in the aquatic environment, it can persist for long periods, altering primary productivity and affecting non-target organisms at different trophic levels. Several studies have demonstrated that atrazine residues disrupt photosynthetic efficiency in microalgae, impair reproduction and growth in zooplankton, and cause endocrine and developmental alterations in fish (Castanha et al., 2023; Tulcan et al., 2021). Despite regulatory restrictions in some regions, atrazine remains extensively detected in surface waters, raising concerns about its long-term ecological risks (Brovini et al., 2023).

Freshwater food webs are highly sensitive to anthropogenic changes, such as agricultural runoff (Dudgeon & Strayer 2024; Song et al., 2026). Atrazine can significantly inhibit algal growth and photosynthesis, promoting damage to the photosystem II reaction center (Sun et al., 2020a, 2020b). In aquatic ecosystems, atrazine can alter the biota, ultimately interfering with the food chains and consequently disrupting the interactions among bacteria, algae, and grazers. In fact, a short-term exposure to a sublethal concentration of atrazine may induce senescence features in microalgal cells, which are the base of aquatic food webs (Esperanza et al., 2017). In sum, the herbicide can be highly toxic to phytoplankton and, thus, may enter higher trophic levels through bioconcentration and food-web transport (Yang et al., 2019).

In recent years, the development of nanoformulated pesticides has been proposed as a strategy to enhance agricultural efficiency while reducing the amount of active ingredients released into the environment. These formulations, usually based on biodegradable polymers or other nanocarriers, are designed to improve herbicide stability, solubility, and controlled release, thereby minimizing off-target contamination (Wu et al., 2021). Since herbicide nanoencapsulation permits a controlled release and may result in an environmental toxicity occurrence decrease, poly(ε-caprolactone)-encapsulated nanoatrazine (nATZ-PCL) has been developed to reduce the adverse impacts of this herbicide on ecosystems and enable more sustainable practices (Kumar et al., 2021).

However, the nanoscale properties that make these systems advantageous in agriculture may also alter their environmental fate, bioavailability, and interaction with aquatic organisms (Ke et al., 2025). Also, different nanoformulations may result in distinct toxicological profiles in *R. subcapitata* (Yang et al., 2026a)

In this way, polymeric nanoencapsulation in poly(ε-caprolactone) has been reported to modify the herbicide’s behavior in water and sediments, potentially influencing its uptake and toxicity in non-target species (Castanha et al., 2023; Sun et al., 2020a, 2020b). Despite these advances, little is known about the bioaccumulation potential and trophic transfer of nATZ-PCL across aquatic food webs, highlighting the need for experimental studies under controlled laboratory conditions.

Once released into aquatic ecosystems, pesticides may enter food webs through bioconcentration, bioaccumulation, and trophic transfer processes, leading to potential biomagnification across successive trophic levels. These mechanisms are well documented for conventional contaminants, including hydrophobic organic compounds and metals, but the behavior of nanostructured formulations remains poorly understood (Ke et al., 2025; Shi et al., 2020). Factors as particle size, surface properties, and polymer composition can affect contaminant bioavailability, uptake by aquatic organisms, and the potential for transfer across trophic levels (Sun et al., 2020a, 2020b; Cardoso e Bufalo et al., 2025). In this context, understanding how nATZ-PCL interacts with aquatic organisms is crucial to assess its potential for bioaccumulation and trophic transfer within food webs. Despite the increasing production and use of nanoformulated pesticides, experimental data describing their accumulation dynamics across aquatic trophic levels remain scarce, representing a significant gap in current ecotoxicological knowledge (Castanha et al., 2023; Chaud et al., 2021).

Therefore, this study aimed to evaluate the bioaccumulation of nATZ-PCL in the microalga *Raphidocelis subcapitata* and to investigate its potential transfer to the zooplanktonic species *Daphnia magna* via both dietary and direct exposure pathways.

## Material and methods

### Synthesis and characterization of nATZ-PCL formulation

Atrazine is encapsulated in poly(ε-caprolactone) (PCL) nanoparticles. This technique protects the active ingredient, reduces environmental leaching, and increases herbicidal efficacy. The atrazine-loaded PCL nanocapsules employed in this study were prepared using the same formulation and interfacial deposition method previously described by Grillo et al. (2012). The physicochemical characterization of this formulation, including particle morphology, encapsulation efficiency (86.7 ± 0.7%), colloidal stability, and in vitro release profile, has been reported in detail previously (Castanha et al., 2023; Takeshita et al., 2021).

nATZ-PCL formulation was produced following a nanoprecipitation approach adapted from Fessi et al. (1989). Initially, an aqueous phase containing polysorbate 80 (2 mg mL^−1^) was prepared. The organic phase was obtained by dissolving myritol (200 mg), atrazine (10 mg), PCL (100 mg), and Span 60 (40 mg) in 30 mL of acetone. Once complete dissolution was achieved, the organic solution was slowly added to the aqueous phase under constant stirring. After 20 min of mixing, the organic solvent was removed by rotary evaporation until the suspension reached a final volume of 10 mL. Under these conditions, the resulting nanoformulation contained atrazine at a final concentration of 1 mg mL^−1^. ATZ analytical standard (Merck): 2-chloro-4-ethylamino-6-isopropylamino-1,3,5-triazine, 1912-24-9 and PCL Sigma-Aldrich 440752 were used for formulation preparation.

The encapsulation efficiency of the atrazine-loaded PCL nanocapsules was previously determined using the ultrafiltration/centrifugation method described by Grillo et al. (2012). This approach is widely employed in polymeric nanoparticle systems because it enables efficient separation of free (non-encapsulated) molecules from the suspension without disrupting the nanocarriers. The suspension is transferred to an ultrafiltration device equipped with a regenerated cellulose membrane (30 kDa MWCO) and centrifuged, allowing only free atrazine molecules to pass through the membrane while the nanoparticles are retained in the upper compartment. The concentration of free atrazine in the filtrate is then quantified by HPLC, and the encapsulation efficiency is calculated from the difference between the total and free atrazine concentrations.

#### Nanoparticle characterization

nATZ-PCL were characterized using size (nm), polydispersity (PDI), and zeta potential (mV) parameters, using dynamic light scattering (ZetaSizer Nano ZS90—Malvern Instruments). The samples were diluted 1000× and measured at 25 °C with the scattered light detected at an angle of 90°.

### nATZ-PCL stability evaluation in a culture medium

The stability of the nATZ-PCL nanoformulation was assessed in liquid culture medium both in the presence and absence of natural organic matter (NOM; Suwannee River, RO isolation—International Humic Substances Society, catalog no. 2R101N). For these assays, a concentration of 10 mg L^−1^ was employed, as this value provided the most reliable conditions for DLS measurements. Because the optimal concentration range is influenced by particle size distribution, the selected concentration was based on the consistently measured hydrodynamic diameter. Additionally, such aqueous matrices can decrease nanoparticle stability, which in turn may increase measurement variability (Hachenberger et al., 2023).

### Experimental design and exposure conditions

Numerous factors can influence atrazine fate, anchored on the chemical, physical, and biological environmental conditions. Soil conditions and precipitation are significant variables influencing atrazine levels in surface water (Stone et al., 2025). Also, climate conditions and agricultural practices promote great variation in atrazine runoff values. More than that, the fate of atrazine can be influenced by extreme rainfall due to climate change (Gonçalves et al., 2023). Thus, the exposure concentrations selected for the experiments were based on the species-specific EC_10_ and EC_25_ values previously determined by our research group for nATZ-PCL using the same test species (Castanha et al., 2023). EC_10_ and EC_25_ were defined as effective concentrations causing 10% and 25% effects, respectively, based on growth inhibition in *R. subcapitata* and immobilization in *D. magna*. These effect levels were chosen to ensure biologically relevant and sublethal exposure conditions. The NOM concentration used in this study was 10 mg L^−1^, representing environmentally relevant conditions (Xiao et al., 2022).

**Fig. 1:**
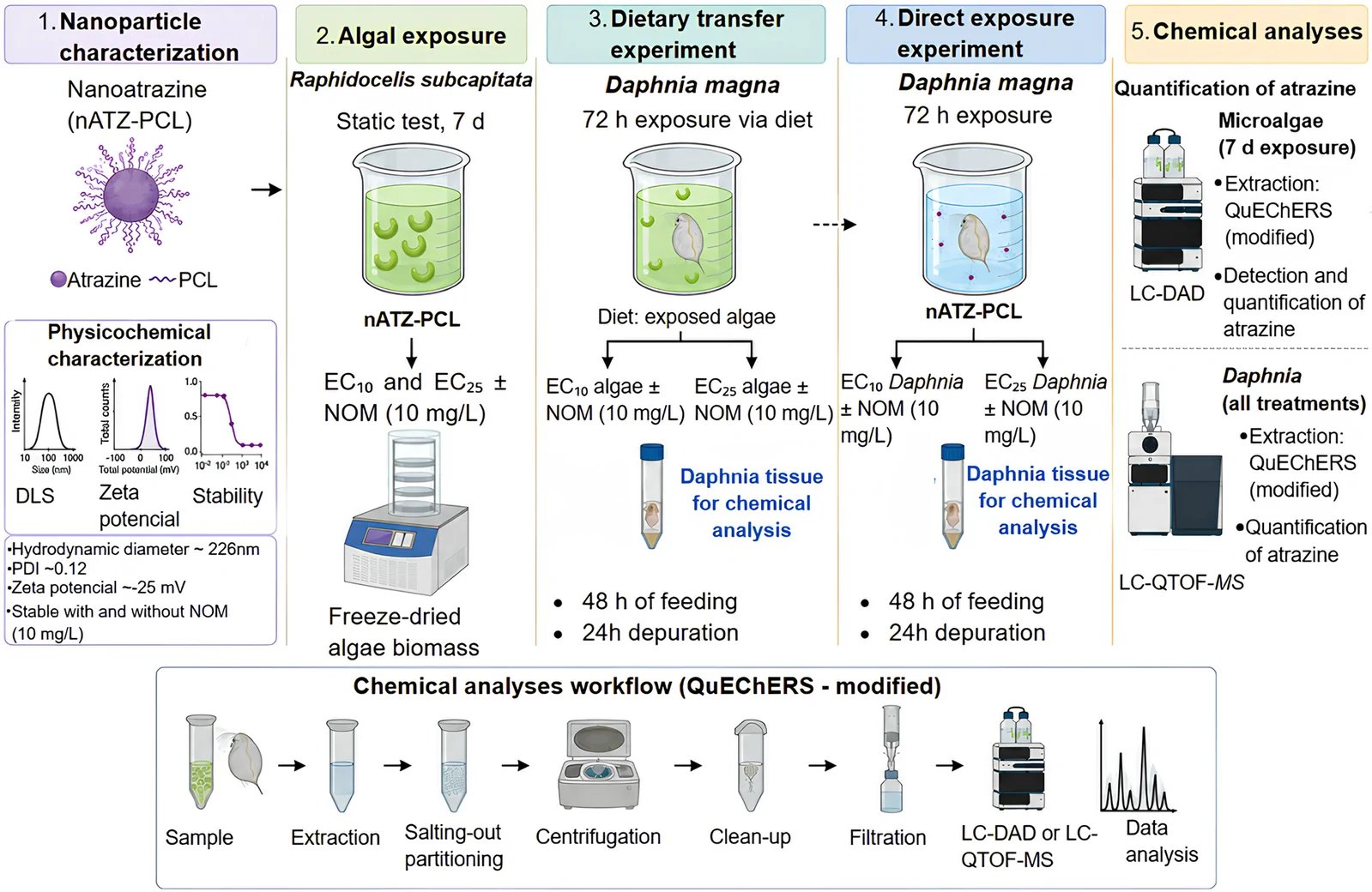
Schematic overview of the experimental design used to evaluate the bioaccumulation and trophic transfer nATZ-PCL, including nanoparticle characterization, exposure assays with *Raphidocelis subcapitata* and *Daphnia magna*, and chemical analyses

For *R. subcapitata*, EC_10_ and EC_25_ values corresponded to 0.040 and 0.103 mg L^−1^ in the absence of NOM, and 0.020 and 0.043 mg L^−1^ in the presence of NOM, respectively. For *D. magna*, EC_10_ and EC_25_ values were 2.05 and 5.69 mg L^−1^ without NOM, and 14.78 and 36.23 mg L^−1^ with NOM, respectively. These values were used to define sublethal exposure conditions for the bioaccumulation and trophic transfer assays. A schematic overview of the experimental workflow is presented in Fig. 1.

#### Bioaccumulation in *Raphidocelis subcapitata*

To assess the uptake of nATZ-PCL by *R. subcapitata*, a bioaccumulation experiment was conducted using two exposure concentrations previously established in acute toxicity tests. The EC_10_ and EC_25_ values for assays performed with and without NOM are summarized in Table 1. Exposures lasted 7 days and were carried out under controlled laboratory conditions at 22 °C, with constant illumination and continuous agitation, using 2-L volumes per treatment. The experiments were repeated on different dates to obtain sufficient algal biomass (1.000 mg) for atrazine quantification by HPLC. Each culture containing 2 L of medium yielded an average of 150 mg of dry biomass, whereas treatments exposed to nATZ-PCL produced approximately 100 mg. Thus, to obtain sufficient dry biomass for atrazine quantification via HPLC, the cultures were repeated 12 times until the necessary quantity of lyophilized algae for the analytical procedures had been accumulated.

**Table 1:** EC_10_ and EC_25_ values and respective upper and lower limits determined for *R. subcapitata* with NOM and without NOM conditions (Castanha et al., 2023)

Condition	EC_10_ (mg L^−1^)	EC_25_ (mg L^−1^)
Without NOM	0.040 (0.01–0.06)	0.103 (0.071–0.125)
With NOM	0.020 (0.00–0.04)	0.043 (0.015–0.066)

At the end of the exposure period, cultures were centrifuged at 10,000 rpm for 10 min, the supernatant was discarded, and the resulting pellet was washed three times to remove superficially adsorbed particles. The samples were then freeze-dried (− 80 °C) and subjected to atrazine extraction using a modified QuEChERS protocol.

The concentration of atrazine accumulated in *R. subcapitata* biomass and the culture medium was also determined after 7 days of exposure to nATZ-PCL at concentrations corresponding to EC_10_ (0.040 mg L^−1^), EC_10_ + NOM (0.020 mg L^−1^), EC_25_ (0.103 mg L^−1^), and EC_25_ + NOM (0.043 mg L^−1^). The samples were lyophilized, subjected to the previously described cleanup procedure, and analyzed by the same HPLC method.

#### Evaluation of nATZ-PCL sedimentation during sample processing

An additional centrifugation assay was performed to evaluate whether the sample processing conditions employed for *R. subcapitata* biomass recovery could induce nATZ-PCL sedimentation and co-precipitation with the cellular pellet. The assay was conducted using nATZ-PCL suspensions at EC_10_ and EC_25_ concentrations, both in the presence and absence of NOM, under the same experimental conditions applied in the algal bioaccumulation assays. Samples collected at exposure times of 0 and 7 days were centrifuged at 10,000 rpm for 10 min, and the formation of visible nATZ-PCL pellets was evaluated.

#### Trophic transfer assessment in *Daphnia magna*

To evaluate the potential trophic transfer of nATZ-PCL, *D. magna* individuals were exposed through two distinct pathways: (i) dietary exposure, using *R. subcapitata* previously exposed to nATZ-PCL, and (ii) direct exposure to nATZ-PCL in the test medium.

For dietary exposure, freeze-dried *R. subcapitata* biomass previously exposed to nATZ-PCL at control, EC_10_, and EC_25_ concentrations, both in the presence and absence of NOM, was used as a food source for *D. magna*. Algal density was standardized across all treatments at 4 × 10^6^ cells mL^−1^, corresponding to approximately 20 mg of algal biomass per 200 mL of test medium.

The experiment was conducted in triplicate, with 100 *D. magna* individuals per Erlenmeyer flask containing 200 mL of reconstituted medium. Organisms were fed for 48 h, with renewal of both the medium and algal suspensions every 24 h to maintain consistent exposure conditions. Following the feeding period, a 24-h depuration phase was performed under clean medium conditions, without feeding or exposure to pre-exposed algae.

At the end of the 72-h experimental period, organisms were collected and gently rinsed three times to remove externally adhered particles. Individuals from each replicate were then pooled and stored at − 80 °C for subsequent analysis.

Comparing the bioaccumulation results with those from direct exposure, experiments were conducted under the same experimental conditions described for the dietary assays. *D. magna* individuals were exposed to nATZ-PCL at concentrations corresponding to their species-specific EC_10_ and EC_25_ values (2.05 and 5.7 mg L^−1^, respectively) directly in the medium.

Organisms were maintained in reconstituted medium and fed with control (non-exposed) *R. subcapitata* at the same density used in the dietary exposure experiments (4 × 10^6^ cells mL^−1^). The exposure duration, renewal procedures, depuration phase, and sampling protocol followed the same conditions described for the dietary assays.

### Extraction and quantification of atrazine by HPLC

#### Atrazine extraction

Atrazine extraction was performed using freeze-dried biomass of *R. subcapitata* (50 mg) and pooled *D. magna* individuals (3 mg), using a micro-QuEChERS procedure. Briefly, 1.0 mL of acetonitrile was added to the sample, followed by the addition of 0.02 g NaCl and 0.08 g anhydrous MgSO_4_. After extraction, a 600-µL aliquot of the organic phase was subjected to dispersive solid-phase extraction (d-SPE) cleanup using 0.01 g primary secondary amine (PSA) and 0.01 g C18 sorbent. Subsequently, a 400-µL aliquot of the purified extract was evaporated to dryness under a gentle stream of nitrogen and reconstituted in 400 µL of the initial mobile phase solution (water: acetonitrile, 40:60 v/v), filtered through a 0.22-µm PVDF membrane before analysis by LC-DAD. To overcome the limitations imposed by the low biomass of pooled *Daphnia magna* samples, atrazine was additionally analyzed by LC–QTOF–MS. The higher sensitivity provided by high-resolution mass spectrometry improved the detection of trace concentrations of atrazine in the biological matrix.

#### *R. subcapitata* LC-DAD analysis

Chromatographic analyses were performed using an LC-DAD system (Shimadzu LC-10ATvp) equipped with a C18 column (150 × 4.6 mm, 5 μm). Separation was achieved using gradient elution with water (A) and acetonitrile (B) as the mobile phase, at a flow rate of 0.6 mL min^−1^ and an injection volume of 20 μL. Detection was carried out at 220 nm, and atrazine was identified based on a retention time of 8.8 min.

In-house validation was performed by evaluating selectivity, limit of detection (LOD), limit of quantification (LOQ), linearity, and extraction efficiency in terms of recovery and precision under repeatability and within-laboratory reproducibility conditions. LOD and LOQ methods were determined using atrazine-spiked blank biomass of *R. subcapitata*. The LOD method (0.0023 µg g^−1^) was defined as the lowest atrazine concentration that could be reliably detected, whereas the LOQ method (0.2 µg g^−1^) was established as the lowest concentration that could be quantified with acceptable recovery (62%) and precision (10%). Linearity was demonstrated over the concentration range of 0.01–0.15 µg mL^−1^, with a coefficient of determination (*r*^2^) greater than 0.99. The instrumental LOD was estimated at 0.007 µg mL^−1^.

#### *D. magna* LC-QTOF-MS analysis

Atrazine was analyzed using an ACQUITY UPLC system (Waters, Milford, MA, USA) coupled to a SYNAPT G1 quadrupole time-of-flight mass spectrometer (QTOF-MS, Waters) equipped with an electrospray ionization (ESI) source operating in positive ionization mode.

Chromatographic separation was performed on an ACQUITY BEH C18 column (2.1 × 100 mm, 1.7 μm) maintained at 40 °C. The mobile phase consisted of (A) water containing 0.1% formic acid and (B) acetonitrile containing 0.1% formic acid, in gradient mode elution. Mass spectrometric acquisition was carried out in ESI positive mode over an *m/z* range of 70–300. The capillary voltage was set at 3.0 kV, the sampling cone voltage at 20 V, the source temperature at 110 °C, and the desolvation temperature at 400 °C. Nitrogen was used as both the cone and desolvation gas at flow rates of 20 and 500 L h^−1^, respectively. Data were acquired in full scan mode. A mass window of 0.05 Da at the exact mass of protonated molecule was applied for the extraction of the ion chromatogram for atrazine. The protonated molecular ion of atrazine ([M + H]^+^, *m/z* 216.1016) was used for quantification by LC-QTOF-MS. Data acquisition and processing were carried out using Mass Lynx software (version 4.1, Waters).

In-house validation was performed following the same procedure described for *R. subcapitata*. The LOD and LOQ methods were 0.3733 and 1.144 µg g^−1^, respectively. Linearity was demonstrated over the concentration range of 0.025–0.125 µg mL^−1^, with a coefficient of determination (*r*^2^) greater than 0.99. The instrumental LOD was estimated at 0.0028 µg mL^−1^.

### Statistical analysis

Statistical analyses were performed using GraphPad Prism software (version 8.0) Data normality and homogeneity of variance were evaluated prior to analysis. Differences among treatments were assessed by one-way analysis of variance (ANOVA) followed by Tukey’s multiple comparisons test. Statistical significance was considered at *p *< 0.05. Results are expressed as mean ± standard deviation (SD).

## Results and discussion

### Physicochemical characterization and stability of nATZ-PCL

The nATZ-PCL presented a mean hydrodynamic diameter of 226 nm, low polydispersity (PDI = 0.12), and a negative zeta potential (− 25 mV), indicating a relatively homogeneous and physically stable nanosuspension.

**Fig. 2:**
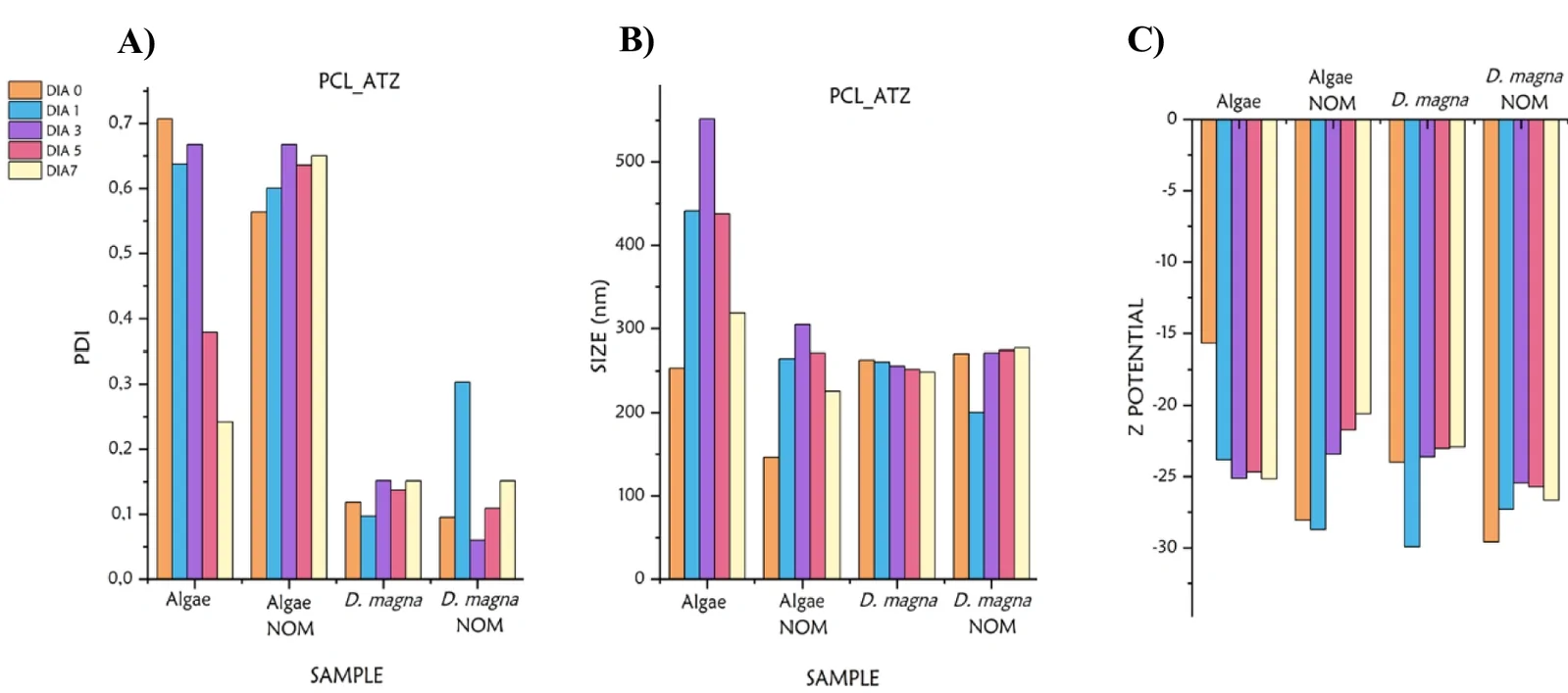
Physicochemical stability of the nATZ-PCL in culture media of Raphidocelis subcapitata and Daphnia magna, in the presence and absence of natural organic matter (NOM), over 7 days of exposure. Variations in **A** polydispersity index (PDI), **B** hydrodynamic diameter, and **C** zeta potential were monitored at different experimental times (days 0, 1, 3, 5, and 7)

Stability assays conducted under environmentally relevant conditions demonstrated that the nATZ-PCL remained relatively stable in most tested media throughout the experimental period, with only minor variations in particle size and polydispersity index (Fig. 2A, B). However, in the culture medium used for *R. subcapitata*, a progressive increase in hydrodynamic diameter and PDI was observed over time, suggesting aggregation processes or surface interactions occurring in this matrix. Variations in zeta potential were comparatively less pronounced across treatments and exposure times (Fig. 2C).

These alterations may be associated with the ionic composition of the culture medium and/or the formation of an abiotic eco-corona mediated by NOM, which can modify nATZ-PCL surface properties and influence their environmental behavior and bioavailability (Liu et al., 2023). These processes may regulate the availability of nATZ-PCL to aquatic organisms, influencing their accumulation in primary producers and the efficiency of transfer across trophic levels.

The increase in hydrodynamic diameter and PDI observed in the algal culture medium suggests that interactions between the nATZ-PCL and medium components promoted aggregation and reduced colloidal homogeneity over time. Similar behavior has been reported for engineered nanomaterials exposed to environmentally relevant matrices, where ionic strength and dissolved organic matter can alter nanoparticle surface chemistry and aggregation dynamics (Hachenberger et al., 2023).

Environmental corona formation mediated by NOM may further influence nanoparticle stability, transport, and biological interactions by modifying surface charge distribution and steric interactions (Xu et al., 2020). Although zeta potential values remained relatively stable throughout the exposure period, the observed increase in particle size indicates that mechanisms other than electrostatic destabilization, such as particle bridging or adsorption-mediated aggregation, may have contributed to the formation of agglomerates in the algal medium.

These physicochemical alterations are particularly relevant because nanoparticle aggregation may directly affect cellular uptake and internalization by aquatic organisms, ultimately influencing bioavailability and trophic transfer efficiency (Liu et al., 2018; Wang et al., 2022).

#### Evaluation of nATZ-PCL sedimentation during sample processing

Considering the aggregation tendency observed in the algal culture medium, a centrifugation stability assay was performed to investigate whether the sample processing conditions could promote nATZ-PCL sedimentation and co-sedimentation with algal biomass. No visible nATZ-PCL pellet formation was observed under any tested condition, including EC_25_ and EC_10_ concentrations in the presence and absence of NOM at both exposure times (0 and 7 days). These results suggest that the centrifugation conditions employed did not induce precipitation or co-sedimentation of the nATZ-PCL with the algal pellet.

### Bioaccumulation in *Raphidocelis subcapitata*

**Fig. 3:**
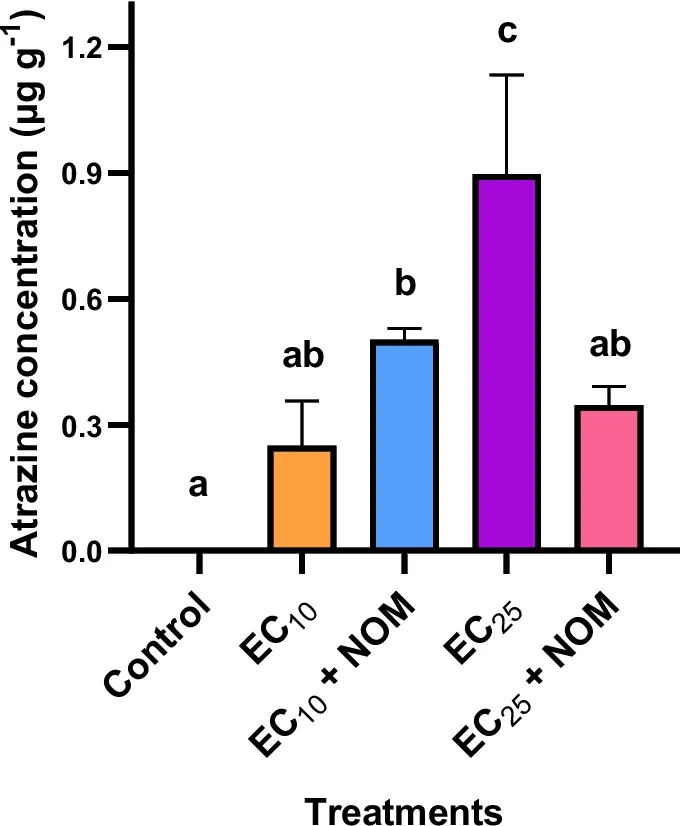
Atrazine concentration detected in *Raphidocelis subcapitata* after exposure to nATZ-PCL under EC_10_ and EC_25_ conditions, in the presence and absence of natural organic matter (NOM). Bars represent mean values ± standard deviation

Atrazine accumulation in *R. subcapitata* varied according to exposure concentration and the presence of NOM (Fig. 3). No atrazine residues were detected in control samples, while experimental treatments showed measurable accumulation levels ranging from 0.25 to 0.89 μg g^−1^.

The highest accumulation was observed in the EC_25_ treatment without NOM, reaching approximately 0.89 μg g^−1^, which was significantly higher than all other treatments (*p *< 0.05). Similar accumulation patterns have previously been reported for atrazine in freshwater primary producers. Nikkilä et al. (2001) demonstrated rapid uptake and bioconcentration of atrazine by periphyton communities, while Kabra et al. (2014) reported atrazine accumulation by the green microalga *Chlamydomonas mexicana* during exposure experiments, resulting in a progressive reduction of herbicide concentrations in the culture medium. However, the data should be examined with caution, since the measured atrazine in algae may reflect both internalized material and some strongly associated particles that were not removed during the sample preparation.

The interaction between NOM and nanoparticles (NPs) can affect behavior and biological interactions, such as bioavailability, biocompatibility, and ecotoxicity toward aquatic organisms (Xu et al., 2020). In the present study, the presence of NOM reduced atrazine accumulation under EC_25_ conditions, resulting in concentrations close to 0.33 μg g^−1^. Similar reductions in nATZ-PCL uptake have been reported following NOM adsorption onto particle surfaces, which can modify colloidal stability, promote eco-corona formation, and alter interactions with biological membranes (Wang et al., 2022; Liu et al., 2023). These processes may decrease nATZ-PCL bioavailability and ultimately limit cellular internalization by primary producers.

Under EC_10_ exposure conditions, the treatment containing NOM exhibited slightly higher accumulation values than the corresponding treatment without NOM, although no statistically significant difference was observed between these groups (*p *> 0.05). This behavior suggests that the influence of NOM on nATZ-PCL bioavailability may depend on exposure concentration and nATZ-PCL–medium interactions.

The observed accumulation patterns may be associated with the physicochemical behavior of the nATZ-PCL in the algal culture medium, particularly considering the aggregation tendency identified during the stability assays. Changes in particle size distribution and surface interactions mediated by NOM can directly affect nATZ-PCL transport, adsorption to cell surfaces, and internalization by microalgae (Liu et al., 2022).

The presence of atrazine was confirmed exclusively in samples of biomass and culture medium exposed to nATZ-PCL—with no corresponding signal detected in the control group—demonstrating the retention of nATZ-PCL by the algal biomass following the experimental period.

These findings show that *R. subcapitata* can accumulate nATZ-PCL under environmentally relevant exposure conditions, highlighting the potential role of primary producers as initial entry points of nanopesticides into aquatic food webs.

### Limited uptake of nATZ-PCL by *Daphnia magna*

Although some degree of NPs bioaccumulation is expected and despite the confirmed accumulation of nATZ-PCL in *R. subcapitata*, atrazine concentrations in *D. magna* remained below the method detection limit (0.373 µg g^−1^) following either dietary exposure with pre-exposed algae or direct exposure in reconstituted medium. Chromatographic responses remained close to the reagent blank and remained below the instrumental LOD, indicating that atrazine, if transferred, was present at concentrations below the analytical detection capability of the method. This suggests limited uptake and trophic transfer under experimental conditions. Limited trophic transfer of nanomaterials has previously been associated with reduced bioavailability and low assimilation efficiency by *D. magna*, as reported for other nanomaterials (Li & Wang, 2023). A limitation of the present study was the relatively small biomass of *D. magna* available for analysis, which resulted in a method detection limit of 0.373 µg g^−1^. Future studies using a larger biomass would enable a lower detection limit and improve the analytical sensitivity, allowing a more definitive determination of whether atrazine trophic transfer occurs at concentrations below the current detection capability.

The great diversity of species in a community has unique biological and physiological traits. This diversity offers a major challenge of environmental risk contaminants assessment, as these traits dictate the chemical uptake and accumulation and, ultimately, its transport along the food web (Brooks et al., 2009). Lee et al. (2015) observed that NP accumulation may be different within organisms. This fact may be due to the physical structure of these organisms and to the available surface area for interaction with NPs. Thus, they can undergo biomagnification depending on the organism’s structure. nATZ-PCL may have adhered to the carapace, locomotor appendages, and NOM, and were not absorbed by daphnia to a detectable level.

The absence of detectable residues suggests that the transfer of nATZ-PCL from the primary producer to the zooplanktonic consumer was insufficient to result in measurable accumulation. One possible explanation is the aggregation behavior observed in the algal culture medium, which may have altered nATZ-PCL bioavailability and reduced uptake efficiency by the organisms (Hachenberger et al., 2023). Since aggregation state was not monitored throughout the exposure period, future investigations using biological assays and in situ physicochemical characterization of nATZ-PCL during exposure will be important to better comprehend the role of changes in nanoparticle behavior in the observed biological responses.

In addition, limited assimilation efficiency and depuration processes may also have contributed to atrazine concentrations remaining below the analytical detection limit, as previously reported for atrazine in *D. magna.* A steady state during uptake by algae and *Daphnia* is reached within hours, while elimination occurs within minutes for algae and within hours for microcrustacean (Nikkilä et al., 2001). Then, it is essential to evaluate the toxicokinetics of nanoformulations in organisms to understand their toxicity, including variations in bioavailability (Wu et al., 2024).

Similar findings were reported by Dionisio et al. (2023), who observed no significant adverse effects in *D. magna* following trophic exposure to nATZ-PCL from algae to zooplankton, suggesting low transfer efficiency across trophic levels. These observations reinforce the hypothesis that nanoencapsulation may alter the environmental behavior and bioavailability of atrazine, potentially reducing its assimilation by primary consumers. It is worth noting that, despite all the hypotheses raised here being plausible, the present experiments do not distinguish the mechanism of the absence of atrazine transfer.

The present findings demonstrate that bioaccumulation in primary producers does not necessarily result in efficient trophic transfer to higher trophic levels. Although *R. subcapitata* accumulated nATZ-PCL, this accumulation did not translate into detectable residues in *D. magna*, suggesting that nATZ-PCL bioavailability and assimilation efficiency may play a critical role in controlling trophic transfer processes. Recent assessments of freshwater food webs have further highlighted that trophic transfer efficiency is highly variable and often constrained by physiological and ecological processes operating at each trophic level. Thus, trophic transfer efficiency varies among trophic levels and may not be the same along the entire food chain (Song et al., 2026). Indeed, a key challenge in ecotoxicology is the potential chemical risks to a wide range of species in the environment based on laboratory toxicity data derived from a limited number of species (Rubach et al., 2011). Concerning nanoparticles’ interactions concomitantly with different materials and pollutants in the environment, organisms are exposed to different transformed ones (Abbas et al., 2020). This limits our ability to generalize the findings to real-world food webs. Meanwhile, an internationally accepted framework for assessing the potential risks associated with nanopesticides is lacking (Rodríguez-Seijo et al., 2025; Yang et al., 2026b).

Therefore, the absence of detectable atrazine residues in *D. magna* does not necessarily indicate a lack of exposure but rather suggests that the amount transferred and retained by the organisms was insufficient to result in measurable accumulation under the experimental conditions employed.

These findings also reveal the complexity of nanopesticide toxicity testing and underscore the need for future studies using more extensive trophic chain protocols to better understand the interactions and ecological risks associated with their exposure (Liu et al., 2018).

## Conclusion

The present study demonstrated that the nATZ-PCL was bioaccumulated by the microalga *R. subcapitata* under environmentally relevant exposure conditions. However, this accumulation did not result in detectable transfer to the zooplanktonic consumer *D. magna*, either through dietary exposure with pre-exposed algae or through direct exposure in reconstituted medium. To better understand the toxicity of nATZ-PCL, future studies should further investigate its physicochemical properties, bioavailability, and toxicokinetic behavior across different trophic levels.

Despite natural aquatic food webs involving greater complexity, the results suggest that factors such as nATZ-PCL aggregation, reduced bioavailability, limited assimilation efficiency, and depuration processes may play an important role in controlling the trophic transfer of nATZ-PCL in aquatic systems under the present experimental conditions in a single producer-consumer interaction.

These findings contribute to the understanding of the environmental behavior of nATZ-PCL and demonstrate that bioaccumulation in primary producers does not necessarily imply efficient transfer across aquatic trophic levels.

## Data Availability

The datasets generated during the current study will be deposited in the Embrapa Research Data Repository (Redape) upon acceptance of the manuscript.
